# Fatty acid composition of the follicular fluid of normal weight, overweight and obese women undergoing assisted reproductive treatment: a descriptive cross-sectional study

**DOI:** 10.1186/1477-7827-12-13

**Published:** 2014-02-05

**Authors:** Sara DM Valckx, Maria Arias-Alvarez, Ingrid De Pauw, Veerle Fievez, Bruno Vlaeminck, Erik Fransen, Peter EJ Bols, Jo LMR Leroy

**Affiliations:** 1 Veterinary Physiology and Biochemistry, University of Antwerp, Universiteitsplein 1, 2610 Wilrijk, Belgium; 2 Dpto. Producción Animal, Facultad de Veterinaria, Universidad Complutense de Madrid, Ciudad Universitaria s/n., 28040 Madrid, Spain; 3 Centre for Reproductive Medicine, ZNA Middelheim, Lindendreef 1, 2020 Antwerpen, Belgium; 4 Laboratory for Animal Nutrition and Animal Product Quality, Ghent University, Proefhoevestraat 10, 9090 Melle, Belgium; 5 StatUA Center for Statistics, University of Antwerp, Prinsstraat 13, 2000 Antwerp, Belgium

**Keywords:** Follicular fluid, Fatty acid, NEFA, BMI, Obesity, ART

## Abstract

**Background:**

It has been well documented that the maturing oocyte is very vulnerable to changes in its micro-environment, the follicular fluid (FF). Recent research has focused on different components within this FF, like hormones, growth factors and metabolites, and how their concentrations are altered by diet and the metabolic health of the mother. It has been proposed that fatty acids (FAs) are potential factors that influence oocyte maturation and subsequent embryo development. However, a thorough study of the specific FF FA composition per lipid fraction and how this may be affected by BMI is currently lacking. Therefore, we investigated the BMI-related concentration of FAs in the phospholipid (PL), cholesteryl-ester (CHE), triglyceride (TG) and non-esterified (NE) lipid fraction in the FF of women undergoing assisted reproductive treatment (ART).

**Methods:**

In this descriptive cross-sectional study, the FF of normal weight (18.5 ≤ BMI < 25.0 kg/m(2), n = 10), overweight (25.0 ≤ BMI < 30.0 kg/m(2), n = 10) and obese (BMI ≥ 30.0 kg/m(2), n = 10) women, undergoing ART, was sampled and analyzed for 23 specific FAs in the PL, CHE, TG and NEFA fraction, using a gas chromatographic analysis method. Differences between BMI-groups were studied by means of univariate general linear models and post hoc Sheffé tests.

**Results:**

Total FA concentrations in the PL and CHE fraction did not differ between BMI groups. Total TG concentrations tended to differ and total NEFA concentrations differed significantly between BMI groups. Interestingly, 42% and 34% of the total FAs was esterified in the PL and CHE fraction, respectively, while only 10% were present in both the TG and NEFA fraction. Only few individual FA concentrations differed in the PL, CHE and TG fraction between BMI groups, whereas abundant BMI-related differences were found in the NEFA fraction.

**Conclusions:**

Our data show that differences in BMI are associated with alterations in the FA composition of the FF, an effect most pronounced in the NEFA fraction. These BMI-related variations could possibly affect granulosa cell viability, oocyte developmental competence and subsequent embryo quality possibly explaining differences in oocyte quality in obese patients described by others.

## Background

Fatty acids (FAs) are important compounds in the micro-environment of the ovarian follicle. Besides their role as cellular energy source, they have important biological functions in cell membrane biogenesis [1,2] and signaling [3]. In addition, they act as precursors for steroids and prostaglandins, which are essential for normal reproductive function [4]. In the follicular fluid (FF), FAs are present in an esterified form [triglycerides (TG), cholesterol esters (CHE) and phospholipids (PL)] or as non-esterified FAs (NEFAs), mainly bound to albumin [5]. Maternal diet can have a profound effect on the FA composition of the FF, which may subsequently affect the FA composition of the cumulus cells and the oocyte [6-8]. Numerous animal studies, mostly in cattle, sheep and rodents, have demonstrated the effect of diet-induced FA changes on ovarian physiology in general and on the quality of the oocyte more specifically [6,8-10]. Dietary induced FA changes or obesogenic diets during oocyte maturation or early embryo development affect oocyte and embryo quality [11,12] and these effects may even have consequences for the offspring’s health [13,14]. However, results of these studies are not always in agreement and difficult to compare as not only the quantity of the FA offered, but also the physiological status, the type of FA (ratios of omega-3, -6 or −9 FAs, number of double bonds, carbon chain length) and the duration of treatment may determine the final FA concentrations in the FF [3]. Besides dietary effects, body condition and metabolic status may also impact on the profile of FAs in the FF [15]. Metabolic disorders such as obesity and type II diabetes, but also a negative energy balance, are associated with upregulated lipolysis, leading to elevated NEFA concentrations in the serum [16], that are reflected in the FF of the dominant follicle [15,17] and could therefore directly affect oocyte quality and metabolism. It has been shown that TG and NEFA concentrations are increased in the FF of obese women subjected to ovum pick-up [18], while such elevated NEFA and/or TG concentrations were negatively associated with human cumulus-oocyte-complex morphology [19] and affected both murine oocyte maturation [18] and bovine embryo quality [20,21]. Other studies investigating the influence of serum or FF FAs on fertility in general and oocyte quality more specifically, are difficult to compare as experimental designs, number and treatment of patients included and the relation between specific FAs studied and fertility outcome parameters often differ [19,22,23]. Additional to the heterogeneity in study design, most research on FAs has focused on the total fat fraction, rather than investigating FA differences in different lipid fraction. This is important though, because the cellular response to FAs possibly depends on the lipid fraction the FA belongs to. For this reason, knowledge on the specific FA composition of each lipid fraction in human FF is essential. In this study, we hypothesized that the FF FA composition may be altered by the woman’s metabolic body condition (as determined by her body mass index, BMI), being a well-documented risk factor for fertility failure [24]. Therefore, we aimed (1) to determine the quantity, the distribution and the relative abundance of FAs in different lipid fractions (PL, CHE, TG and NEFA) in the FF of the dominant follicle of normal weight, overweight and obese women, undergoing assisted reproductive treatment (ART) and (2) to define how this FA distribution is affected by BMI in these women. To our knowledge, this is the first time that a detailed description of FA concentrations and abundance in different lipid fractions in the FF of normal weight, overweight and obese women, undergoing ART is provided. This study further discusses potential implications of BMI-related FF FA changes on oocyte and embryo quality.

## Methods

### Patients and follicular fluid sampling

This descriptive cross-sectional study was approved by the ethical committees of the University of Antwerp (UA A09-04) and the ZNA Middelheim Hospital (EC 3352). Women seeking assisted reproductive services were recruited (voluntary informed consent) at the Center for Reproductive Medicine of the ZNA Middelheim Hospital. Patients were treated with a long agonist protocol, followed by ovum pick-up, *in vitro* fertilization (IVF) or intra-cytoplasmic sperm injection (ICSI) and embryo transfer on Day 3 of culture as described by Valckx et al. [17]. Height (m) and weight (kg) of the patients were used to calculate the body mass index (BMI) score (kg/m^2^). Ten patients were randomly selected for each BMI group out of a population of approximately 100 women from our previous study [17]. The single criterion for this selection was 18.5 ≤ BMI < 25.0 kg/m^2^ for the normal weight group, 25.0 ≤ BMI < 30 kg/m^2^ for the overweight group and BMI ≥ 30.0 kg/m^2^ for the obese group, according to the standards of the World Health Organization [25]. Male and female subfertility etiologies were documented and further patient specific data included the age of the patient (years), IVF attempt number, ratio ICSI/IVF, dosage of gonadotropins used (IU), maximal serum estradiol values (pg/ml), the number of oocytes aspirated upon oocyte retrieval, the number of fertilized oocytes (presenting with 2 pronuclei, 2PN), the number of embryos produced, the number of top quality embryos, the number of embryos transferred and the number of live births. Besides, several ratios were calculated as surrogate markers for oocyte quality: percentage of 2PN (n. 2PN /n. oocytes), percentage of embryos (n. embryos /n. oocytes), percentage of top quality embryos (n. top quality embryos /n. of oocytes) and percentage of embryos and top quality embryos developing from fertilized oocytes (n. embryos /n. 2PN and n. top quality embryos /n. 2PN, respectively). A top quality embryo was defined as an embryo with: (i) four or five blastomeres on Day 2, and seven or more blastomeres on Day 3 (ii) 20% fragmentation or less on Day 3 and (iii) no multi-nucleated blastomeres ever as described by Van Royen *et al.*[26].

For each patient, the FF of the largest and first punctured follicle was recovered during the oocyte retrieval procedure by means of a transvaginal follicular aspiration. Only patients with a blood free aspirate of a follicle ≥ 18 mm were considered for inclusion in the study. The FF samples were cooled immediately after aspiration and were transported on ice within 2 h of collection. At the laboratory, FF samples were centrifuged (1500 g, 10 min) and the supernatant was stored at −80°C until all 30 samples were analyzed for various FAs by means of gas chromatography.

### Gas chromatographic analysis of follicular fluid

Lipids in the FF supernatant were extracted with methyl-tert-butyl-ether as described by Matyash *et al.*[27]. Lipid fractions were separated using SPE-columns [28,29]. Total plasma lipid extracts were dissolved in chloroform (1.0 ml) and applied to an aminopropyl silica column (pasteur pipette containing 100 mg aminopropyl silica gel) under gravity. Cholesteryl-esters and TGs were eluted with chloroform (1.0 ml and 0.5 ml), combined, dried under N_2_ and dissolved in 1.0 ml hexane. Non-esterified FAs were eluted with diethyl ether/acetic acid (100:2; 1.0 ml and 0.5 ml) and PLs with 1 ml methanol/chloroform (6:1) followed by 0.5 ml 0.05 M sodium acetate in methanol/chloroform (6:1). Cholesteryl-esters and TGs were further separated on a pre-packed 100 mg aminopropyl column (Varian). The CHE and TG fractions were loaded in 1 ml hexane and the CHE fraction was eluted with hexane (1.0 ml and 0.5 ml). Triglycerides were eluted with hexane/chloroform/ethyl acetate (100:5:5; 1.0 ml and 0.5 ml). Fatty acids in lipid extracts were methylated using a basic followed by an acid methylation step. Toluene (2 ml) containing the internal standard (C13:0) and methanolic NaOH (2 ml) was added and the mixture was incubated at 70°C (60 min) followed by 30 min at 50°C after addition of methanolic HCl (3 ml), prepared by dissolving 10 ml acetyl chloride in 50 ml methanol. Fatty acid methyl esters (FAME) were extracted with hexane. Fatty acids in the TG fraction were methylated as described by Chouinard *et al.*[30] whereas NEFAs were methylated by an acid methylation step only. Fatty acids in PL and CHE were methylated using a basic followed by an acid methylation step.

Composition analyses of the FAs were conducted using a gas chromatograph (HP 7890A, Agilent Technologies, Diegem, Belgium) equipped with a 75-m SP-2560™ capillary column (i.d., 0.18 mm, film thickness, 0.14 μm; Supelco Analytical, Bellefonte, PA) and a flame ionization detector. The oven temperature program was 70°C before being raised to 175°C (50°C/min) for 13 min, after which it was raised to 215°C (5°C/min) for 25 min. The inlet temperature was 250°C and the detector temperature 255°C. Various control materials were used: BR2/BR3, (Larodan Fine Chemicals, Malmö, Sweden), Supelco® 37 (Supelco Analytical, Bellefonte, PA), PUFA-3 (Matreya LLC, Pleasant Gap, PA) and GLC463 (NU-CHEK-PREP Inc., Elysian, MN, USA). Fatty acids for which no standards were commercially available were identified by order of elution according to Precht *et al.*[31] and Kramer *et al.*[32].

The FA analysis generated data on the concentrations of lauric acid (12:0), myristic acid (14:0), pentadecaenoic acid (15:0), palmitic acid (16:0), palmitoleic acid n-7 (16:1 n-7), palmitoleic acid n-9 (16:1 n-9), margaric acid (17:0), stearic acid (18:0), oleic acid (18:1 n-9), vaccenic acid n-11 (18:1 n-11), linoleic acid (18:2 n-6), arachidic acid (20:0), gamma-linolenic acid (18:3 n-6), alfa-linolenic acid (18:3 n-3), eicosenoic acid (20:1), eicosadienoic acid (20:2), di-homo-gamma-linolenic acid (20:3 n-6), arachidonic acid (20:4 n-6), eicosapentaenoic acid (20:5 n-3), docosatetraenoic acid (22:4 n-6), docosapentaenoic acid n-6 (22:5 n-6), docosapentaenoic acid n-3 (22:5 n-3), docosahexaenoic acid (22:6 n-3). For each FA measurement, both the absolute (μmol/l) and the relative concentration (mol%) was determined. Fatty acids were attributed to the PL, TG, CHE or NEFA fraction.

### Statistical analyses

All statistical analyses were performed with PASW 18.0 (for Windows, Chicago, IL, USA) or R 2.13.1 [33]. Several statistical hypothesis tests were carried out to study the relation between patient characteristics and reproductive outcome parameters, with the type of statistical test depending on the nature of the outcome parameter. Counted numbers were analyzed using Quasipoisson regression (mean IVF attempt number, n. oocytes, n. 2PN, n. embryos, n. top quality embryos), with an F-test as described by Agresti [34]. Numeric variables were analyzed using linear regression (age, gonadotropin dose, maximal estradiol values) and binary outcomes were analyzed using logistic regression (live birth, percentage of 2PN, percentage of embryos and top quality embryos, calculated on the n. of oocytes and on the n. of 2PN). The association between the number of embryos transferred and BMI was tested by means of a Kruskal – Wallis test. Differences in the IVF/ICSI ratio were studied by means of a Student’s t test. In these tests, BMI was entered as a continuous variable. Univariate general linear models, with post hoc Sheffé tests, were used to study differences in FA concentrations within lipid fractions and between BMI groups. In these latter tests, BMI was entered as a categorical variable. The same linear model was used to study the FF FA composition between PCOS and non-PCOS patients. Statistical significance was set at *P* < 0.05. Fatty acid concentrations (absolute or relative) were log transformed in case their distribution was not normal. Data are presented as means ± standard deviation (SD).

## Results

### Patient characteristics

Patient characteristics are presented in Table 1. Our data showed that increasing BMI was significantly associated with a higher dosage of gonadotropins administered (IU, *P* = 0.04). Also, increasing BMI was associated with fewer 2PNs (*P* < 0.01) and lower 2PN percentages (*P* = 0.03). There was no association between BMI and age, mean IVF attempt number, IVF/ICSI ratio, the number of oocytes aspirated, the number of embryos, the percentage of embryos, the number of top quality embryos, the percentage of top quality embryos, the ratios ‘n. embryos /n. 2PNs’ and ‘n. top quality embryos /n. 2PNs’, the number of embryos transferred, the number of live births and maximal estradiol values. As presented in Table 1, PCOS patients were not excluded from the study. This choice was made based on the observation that there was no significant difference in FF FA concentrations in the different lipid fractions between PCOS and non-PCOS patients. Data from our previous study, from which the cohort in this study is a subset, also showed that primary infertility cause did not affect FF composition [17].

**Table 1:** Patient information according to BMI class

	**18.5 ≤ BMI < 25.0**	**25.0 ≤ BMI < 30.0**	**BMI ≥ 30.0**	**Statistical method**	***P*-value**
	**(n = 10)**	**(n = 10)**	**(n = 10)**		
*Age (years)*	31.5 ± 4.67	32.7 ± 4.3	35.0 ± 6.8	Linear regression	NS
*Body Weight (kg)*	61.6 ± 4.7	75.6 ± 11.8	95.5 ± 11.4	NA	
*BMI (kg/m*^*2*^*)*	21.5 ± 0.3	28.1 ± 1.5	34.2 ± 2.4	NA	
*Mean IVF attempt number*	1.3 ± 0.7	2.0 ± 1.3	2.2 ± 1.5	Quasipoisson regression	NS
*ICSI/IVF*	5/5	2/8	5/5	Student’s t test	NS
*Gonadotropins administered (IU)*	1793 ± 554	2100 ± 1034	2645 ± 933	Linear regression	0.04
*Infertility cause (n)*				NA	
*Idiopathic*	8	7	7		
*PCOS*	1	1	2		
*Endometriosis*	0	2	0		
*Tubal*	1	0	1		
*No. oocytes*	9.1 ± 3.6	7.2 ± 2.9	6.7 ± 4.8	Quasipoisson regression	NS
*No. 2PN*	6.5 ± 2.2	4.5 ± 1.1	3.5 ± 2.1	Quasipoisson regression	< 0.01
*No. 2PN/No. oocytes (%)*	73.1 ± 12.0	67.3 ± 18.8	61.1 ± 24.9	Logistic regression	0.03
*No. embryos*	2.3 ±1.2	1.5 ± 0.5	1.8 ± 0.6	Quasipoisson regression	NS
*No. embryos/No. oocytes (%)*	30.2 ± 22.3	22.8 ± 9.1	42.0 ± 32.1	Logistic regression	NS
*No. embryos/No. 2PN (%)*	39.8 ± 24.9	35.6 ± 16.5	64.2 ± 28.2	Logistic regression	NS
*No. top quality embryos*	2.0 ± 1.5	0.8 ± 0.9	1.0 ± 1.1	Quasipoisson regression	NS
*No. top quality embryos/No. oocytes (%)*	26.9 ± 25.4	11.4 ± 15.2	19.4 ± 30.1	Logistic regression	NS
*No. top quality embryos/No. 2PN (%)*	34.5 ± 29.8	19.6 ± 24.8	30.2 ± 33.1	Logistic regression	NS
*No. embryos transferred*	1.1 ± 0.3	1.3 ± 0.5	1.5 ± 0.5	Quasipoisson regression	NS
*No. live births*	3	2	5	Logistic regression	NS
*Maximal E*_*2*_*values (pg/ml)*	1571 ± 445	1399 ± 769	1505 ± 928	Linear regression	NS

The statistical method used and the associated *P*-value are presented in the last two columns.

Data are presented as mean ± standard deviation. BMI: body mass index, IVF: *in vitro* fertilization, PCOS: poly cystic ovarian syndrome, 2PN: presence of 2 pronuclei, E_2_: estradiol, NS: not significant (*P* > 0.05), NA: Not applicable.

### Total FA concentrations in the FF per lipid fraction

The distribution of FAs in the different lipid fractions in FF from normal weight, overweight and obese women is presented in Table 2. In obese women, NEFA concentrations were elevated (*P* < 0.05) compared to normal weight and overweight women. The concentration of TG tended to be higher in overweight women, compared to normal weight women (*P* = 0.1). Total FA concentrations did not differ between BMI groups (Table 2). It is noteworthy that the individually measured FA concentrations, in all the lipid fractions, had a relatively high standard deviation. Forty-one up to 45% of the defined FAs in the FF could be attributed to the PL fraction, 32-36% to the CHE fraction, 8-13% to the TG fraction and 9-11% to the NEFA fraction and this distribution did not differ between BMI groups. Total concentrations, presented in Table 2, also included measures of unknown FAs (accounting for approximately 5% of the total FA concentration).

**Table 2:** Fatty acids in follicular fluid of normal weight, overweight and obese women

**Fat fraction**	**18.5 ≤ BMI < 25.0 (n = 10)**	**25.0 ≤ BMI < 30.0 (n = 10)**	**BMI ≥ 30.0 (n = 10)**	***P*-value**
Phospholipids (μmol/l)	1165.57 ± 180.55	1147.93 ± 247.63	1199.95 ± 296.46	NS
Cholesteryl esters (μmol/l)	920.04 ± 196.64	891.22 ± 117.35	1067.29 ± 269.40	NS
Triglycerides (μmol/l)	215.91 ± 57.89	355.39 ± 226.44	244.78 ± 74.65	0.1
NEFAs (μmol/l)	221.67 ± 23.00^a^	245.55 ± 35.98^a^	315.53 ± 82.68^b^	< 0.05
Total concentration (μmol/l)	2598.56 ± 422.68	2769.88 ± 477.07	2931.80 ± 684.58	NS

Total fatty acid concentrations (μmol/l) in different lipid fractions in the follicular fluid of normal weight (18.5 ≤ BMI < 25), overweight (25.0 ≤ BMI < 30) and obese (BMI ≥ 30.0) women.

Data are presented as means ± standard deviation. *P*-values of the univariate general linear model analyses are presented in the last column. ^ab^Data with a different superscript differ significantly (*P* < 0.05), NS: Not significant (*P* > 0.05), BMI: body mass index, NEFAs: non-esterified fatty acids.

### BMI dependent distribution of FAs in different lipid classes

#### Total fat extract

The most abundant FAs in the FF were 16:0, 18:0, 18:1 n-9, 18:2 n-6 and 20:4 n-6. No significant differences could be found for neither absolute nor relative concentrations of the individual FAs between BMI groups.

Individual FA concentrations in the different lipid fractions are presented in Table 3.

**Table 3:** Fatty acid concentrations in different lipid fractions

						
**Phopholipid**	**Absolute concentrations (μM)**			**Relative concentrations (mol%)**		
	**Normal weight**	**Overweight**	**Obese**	**Normal weight**	**Overweight**	**Obese**
***12:0***	0,0 ± 0,0	0,2 ± 0,3	0,2 ± 0,6	0,0 ± 0,0	0,0 ± 0,0	0,0 ± 0,0
***14:0***	4,1 ± 1,1	4,6 ± 1,8	4,1 ± 1,1	0,3 ± 0,1	0,4 ± 0,1	0,3 ± 0,1
***15:0***	2,7 ± 0,6	2,7 ± 0,7	2,4 ± 0,4	0,2 ± 0,0	0,2 ± 0,0	0,2 ± 0,0
***16:0***	359,4 ± 53,1	360,9 ± 92,0	383,5 ± 129,0	30,9 ± 1,1	31,3 ± 1,7	31,5 ± 2,6
***16:1 n-7***	1,7 ± 0,3	1,7 ± 0,4	1,6 ± 0,2	0,1 ± 0,0	0,2 ± 0,0	0,1 ± 0,0
***16:1 n-9***	5,8 ± 1,8	6,2 ± 2,8	5,5 ± 1,8	0,5 ± 0,1	0,5 ± 0,1	0,5 ± 0,1
***17:0***	4,3 ± 0,8	4,3 ± 0,8	4,2 ± 0,8	0,4 ± 0,0	0,4 ± 0,0	0,4 ± 0,1
***18:0***	160,0 ± 26,6	160,6 ± 24,2	164,2 ± 25,0	13,7 ± 1,0	14,2 ± 1,5	14,0 ± 2,1
***18:1 n-9***	104,2 ± 16,9	91,4 ± 26,4	95,1 ± 25,6	9,0 ± 0,7^a^	7,9 ± 0,8^b^	7,9 ± 0,7^b^
***18:1 n-11***	17,8 ± 2,9	16,9 ± 4,4	15,8 ± 4,0	1,5 ± 0,2	1,5 ± 0,2	1,3 ± 0,2
***18:2 n-6***	218,1 ± 39,7	191,1 ± 36,7	190,1 ± 26,1	18,7 ± 1,7	16,8 ± 2,2	16,3 ± 2,7
***20:0***	0,9 ± 0,1	0,8 ± 0,1	0,8 ± 0,1	0,1 ± 0,0	0,1 ± 0,0	0,1 ± 0,0
***18:3 n-6***	0,3 ± 0,1	0,4 ± 0,2	0,4 ± 0,1	0,0 ± 0,0	0,0 ± 0,0	0,0 ± 0,0
***18:3 n-3***	2,7 ± 0,7	2,4 ± 0,8	2,1 ± 0,6	0,2 ± 0,1	0,2 ± 0,1	0,2 ± 0,1
***20:1***	2,3 ± 0,3	2,3 ± 0,5	2,2 ± 0,4	0,2 ± 0,0	0,2 ± 0,0	0,2 ± 0,0
***20:2***	4,9 ± 1,3	5,3 ± 1,2	5,0 ± 0,9	0,4 ± 0,1	0,5 ± 0,1	0,4 ± 0,1
***20:3 n-6***	40,1 ± 14,2	45,8 ± 8,5	48,7 ± 12,5	3,4 ± 0,7^a^	4,0 ± 0,6^b^	4,1 ± 0,6^b^
***20:4 n-6***	126,2 ± 19,4	132,2 ± 34,4	154,2 ± 63,4	10,9 ± 0,7	11,5 ± 1,6	12,5 ± 2,1
***20:5 n-3***	9,2 ± 3,0	12,3 ± 7,2	12,7 ± 5,7	0,8 ± 0,2	1,0 ± 0,5	1,0 ± 0,4
***22:4 n-6***	4,8 ± 0,7	5,1 ± 1,3	5,3 ± 2,0	0,4 ± 0,0	0,4 ± 0,1	0,4 ± 0,1
***22:5 n-6***	3,6 ± 1,0	3,8 ± 1,4	3,5 ± 1,4	0,3 ± 0,1	0,3 ± 0,1	0,3 ± 0,1
***22:5 n-3***	12,2 ± 1,6	12,6 ± 3,4	11,5 ± 3,4	1,1 ± 0,1	1,1 ± 0,1	1,0 ± 0,2
***22:6 n-3***	48,6 ± 11,0	54,7 ± 18,1	57,9 ± 19,7	4,2 ± 0,7	4,7 ± 0,9	4,8 ± 0,9
***unknown***	31,8 ± 9,6	29,7 ± 8,5	29,1 ± 7,7	-	-	-
***total***	1165,6 ± 180,6	1147,9 ± 247,6	1200,0 ± 296,5	-	-	-
**Cholesteryl ester**	**Absolute concentrations (μM)**			**Relative concentrations (mol%)**		
	**Normal weight**	**Overweight**	**Obese**	**Normal weight**	**Overweight**	**Obese**
***12:0***	19,6 ± 14,7	23,1 ± 15,0	22,7 ± 14,4	2,1 ± 1,6	2,6 ± 2,1	1,8 ± 1,4
***14:0***	15,9 ± 7,9	17,7 ± 8,5	18,1 ± 8,0	1,7 ± 0,8	2,0 ± 1,7	0,9 ± 0,7
***15:0***	2,2 ± 0,7	2,0 ± 0,7	2,2 ± 0,6	0,2 ± 0,1	0,2 ± 0,2	0,0 ± 0,1
***16:0***	104,8 ± 21,8	103,9 ± 20,7	131,3 ± 45,3	11,4 ± 0,3	11,6 ± 12,1	1,1 ± 1,1
***16:1 n-7***	4,1 ± 1,1	4,0 ± 0,9	4,7 ± 1,8	0,4 ± 0,0	0,4 ± 0,4	0,1 ± 0,1
***16:1 n-9***	19,2 ± 7,9	21,1 ± 10,3	25,0 ± 16,5	2,0 ± 0,5	2,3 ± 2,2	1,0 ± 1,0
***17:0***	1,2 ± 0,2	1,3 ± 0,2	1,4 ± 0,5	0,1 ± 0,0	0,2 ± 0,1	0,0 ± 0,0
***18:0***	7,7 ± 1,5	8,1 ± 0,7	12,9 ± 13,7	0,8 ± 0,1	0,9 ± 1,1	0,1 ± 0,9
***18:1 n-9***	163,1 ± 34,4	147,6 ± 24,6	188,1 ± 66,4	17,8 ± 0,9	16,5 ± 17,3	1,4 ± 1,9
***18:1 n-11***	11,2 ± 2,0	10,3 ± 2,6	12,2 ± 4,2	1,2 ± 0,1	1,1 ± 1,1	0,2 ± 0,1
***18:2 n-6***	415,0 ± 102,5	386,7 ± 52,4	437,6 ± 65,9	45,0 ± 3,6	43,5 ± 42,0	4,0 ± 5,7
***20:0***	0,0 ± 0,0	0,0 ± 0,0	0,0 ± 0,0	0,0 ± 0,0	0,0 ± 0,0	0,0 ± 0,0
***18:3 n-6***	3,7 ± 2,1	3,9 ± 1,3	5,1 ± 2,2	0,4 ± 0,1	0,4 ± 0,5	0,1 ± 0,2
***18:3 n-3***	4,2 ± 1,3	4,3 ± 1,2	5,5 ± 4,4	0,5 ± 0,1	0,5 ± 0,5	0,1 ± 0,3
***20:1***	0,0 ± 0,0	0,0 ± 0,0	0,2 ± 0,7	0,0 ± 0,0	0,0 ± 0,0	0,0 ± 0,0
***20:2***	0,0 ± 0,0	0,0 ± 0,0	0,1 ± 0,4	0,0 ± 0,0	0,0 ± 0,0	0,0 ± 0,0
***20:3 n-6***	7,3 ± 2,8^a^	8,5 ± 1,6^ab^	10,1 ± 2,3^b^	0,8 ± 0,2^a^	1,0 ± 1,0^ab^	0,2 ± 0,1^b^
***20:4 n-6***	65,8 ± 14,4	71,6 ± 18,6	94,7 ± 39,7	7,2 ± 0,8	8,0 ± 8,8	1,4 ± 2,1
***20:5 n-3***	6,5 ± 2,3	8,4 ± 4,9	10,4 ± 5,4	0,7 ± 0,2	0,9 ± 1,0	0,5 ± 0,4
***22:4 n-6***	0,0 ± 0,0	0,0 ± 0,0	0,0 ± 0,0	0,0 ± 0,0	0,0 ± 0,0	0,0 ± 0,0
***22:5 n-6***	0,1 ± 0,2	0,1 ± 0,2	0,2 ± 0,3	0,0 ± 0,0	0,0 ± 0,0	0,0 ± 0,0
***22:5 n-3***	1,3 ± 0,3	1,2 ± 0,1	1,0 ± 0,3	0,1 ± 0,1	0,1 ± 0,1	0,0 ± 0,0
***22:6 n-3***	5,6 ± 1,6	6,6 ± 2,1	8,0 ± 2,8	0,6 ± 0,1	0,7 ± 0,8	0,2 ± 0,2
***unknown***	61,6 ± 8,0	60,7 ± 5,5	75,9 ± 37,6	-	-	-
***total***	920,0 ± 196,6	891,2 ± 117,4	1067,3 ± 269,4	-	-	-
**Triglyceride**	**Absolute concentrations (μM)**			**Relative concentrations (mol%)**		
	**Normal weight**	**Overweight**	**Obese**	**Normal weight**	**Overweight**	**Obese**
***12:0***	2,0 ± 0,6	3,3 ± 3,1	2,4 ± 0,5	1,0 ± 0,3	0,9 ± 0,3	1,0 ± 0,2
***14:0***	5,6 ± 2,1	9,8 ± 8,1	6,8 ± 2,8	2,6 ± 0,7	2,7 ± 0,6	2,8 ± 0,6
***15:0***	1,1 ± 0,2	1,4 ± 0,6	1,1 ± 0,3	0,5 ± 0,1	0,4 ± 0,1	0,5 ± 0,1
***16:0***	51,4 ± 15,4	90,7 ± 70,1	60,8 ± 21,9	23,6 ± 1,2	24,4 ± 2,5	24,5 ± 2,4
***16:1 n-7***	2,2 ± 0,6	3,2 ± 1,7	2,3 ± 0,4	1,0 ± 0,1	1,0 ± 0,2	1,0 ± 0,3
***16:1 n-9***	6,5 ± 3,3	11,8 ± 11,6	7,1 ± 2,9	2,9 ± 0,8	3,1 ± 0,8	2,9 ± 0,5
***17:0***	0,8 ± 0,2	1,2 ± 0,7	0,9 ± 0,3	0,4 ± 0,0	0,4 ± 0,1	0,4 ± 0,1
***18:0***	11,2 ± 4,3	19,5 ± 14,7	15,2 ± 10,5	5,2 ± 1,1	5,5 ± 2,0	5,9 ± 2,1
***18:1 n-9***	76,7 ± 22,9	120,9 ± 74,0	83,4 ± 29,3	35,3 ± 1,9	34,1 ± 2,3	33,9 ± 1,9
***18:1 n-11***	4,2 ± 1,5	7,4 ± 5,0	4,7 ± 1,8	1,9 ± 0,2	2,1 ± 0,3	1,9 ± 0,2
***18:2 n-6***	27,2 ± 7,7^a^	46,8 ± 26,0^b^	30,5 ± 8,0^ab^	12,6 ± 1,4	13,6 ± 1,9	12,8 ± 2,5
***20:0***	0,3 ± 0,0	0,4 ± 0,2	0,3 ± 0,1	0,1 ± 0,0	0,1 ± 0,0	0,1 ± 0,0
***18:3 n-6***	0,4 ± 0,2	0,7 ± 0,5	0,5 ± 0,3	0,2 ± 0,0	0,2 ± 0,1	0,2 ± 0,1
***18:3 n-3***	2,4 ± 0,9	4,4 ± 3,3	3,0 ± 2,0	1,2 ± 0,3	1,2 ± 0,5	1,2 ± 0,6
***20:1***	0,7 ± 0,2	1,4 ± 1,0	0,8 ± 0,5	0,3 ± 0,1	0,4 ± 0,1	0,3 ± 0,1
***20:2***	0,7 ± 0,2	1,0 ± 0,5	0,7 ± 0,2	0,4 ± 0,1	0,3 ± 0,1	0,3 ± 0,1
***20:3 n-6***	0,4 ± 0,1	0,9 ± 0,8	0,5 ± 0,1	0,2 ± 0,0	0,2 ± 0,1	0,2 ± 0,1
***20:4 n-6***	2,1 ± 0,7	4,2 ± 3,5	3,5 ± 1,7	1,0 ± 0,2	1,3 ± 0,6	1,5 ± 0,7
***20:5 n-3***	0,6 ± 0,2	1,2 ± 0,9	0,8 ± 0,4	0,3 ± 0,1	0,3 ± 0,1	0,3 ± 0,1
***22:4 n-6***	0,5 ± 0,2	0,6 ± 0,5	0,4 ± 0,2	0,2 ± 0,1	0,2 ± 0,1	0,2 ± 0,1
***22:5 n-6***	0,0 ± 0,0	0,0 ± 0,0	0,0 ± 0,1	0,0 ± 0,0	0,0 ± 0,0	0,0 ± 0,0
***22:5 n-3***	0,4 ± 0,1	0,8 ± 0,9	0,4 ± 0,1	0,2 ± 0,0	0,2 ± 0,1	0,2 ± 0,0
***22:6 n-3***	0,5 ± 0,2^a^	1,9 ± 2,9^b^	1,0 ± 0,4^ab^	0,2 ± 0,1	0,4 ± 0,3	0,4 ± 0,2
***unknown***	17,9 ± 3,6	21,8 ± 7,9	17,7 ± 4,1	-	-	-
***total***	215,9 ± 57,9	355,4 ± 226,4	244,8 ± 74,7	-	-	-
**NEFA**	**Absolute concentrations (μM)**			**Relative concentrations (mol%)**		
	**Normal weight**	**Overweight**	**Obese**	**Normal weight**	**Overweight**	**Obese**
***12:0***	2,0 ± 0,6	2,6 ± 1,4	2,7 ± 0,9	0,9 ± 0,3	1,0 ± 0,6	0,9 ± 0,3
***14:0***	4,6 ± 0,6^a^	5,1 ± 1,0^ab^	6,4 ± 1,7^b^	2,1 ± 0,2	2,1 ± 0,2	2,0 ± 0,2
***15:0***	0,5 ± 0,1^a^	0,6 ± 0,1^ab^	0,7 ± 0,2^b^	0,2 ± 0,0	0,2 ± 0,0	0,2 ± 0,1
***16:0***	51,4 ± 6,4^a^	55,7 ± 7,2^a^	70,3 ± 17,1^b^	23,2 ± 1,6	22,8 ± 1,6	22,5 ± 2,1
***16:1 n-7***	0,8 ± 0,1^a^	1,0 ± 0,2^ab^	1,4 ± 0,5^b^	0,4 ± 0,0	0,4 ± 0,0	0,4 ± 0,1
***16:1 n-9***	6,3 ± 1,5^a^	7,7 ± 1,7^ab^	10,2 ± 3,8^b^	2,8 ± 0,6	3,1 ± 0,4	3,2 ± 0,6
***17:0***	0,6 ± 0,1^a^	0,7 ± 0,1^ab^	0,8 ± 0,3^b^	0,3 ± 0,0	0,3 ± 0,0	0,3 ± 0,1
***18:0***	19,7 ± 3,6^a^	20,3 ± 2,9^ab^	24,8 ± 5,6^b^	8,9 ± 1,4	8,3 ± 0,9	8,1 ± 1,5
***18:1 n-9***	68,1 ± 8,3^a^	76,9 ± 13,5^ab^	102,5 ± 34,1^b^	30,7 ± 1,8	31,3 ± 1,8	32,1 ± 3,3
***18:1 n-11***	5,8 ± 0,9^a^	6,5 ± 1,6^ab^	9,0 ± 3,0^b^	2,6 ± 0,3	2,6 ± 0,4	2,8 ± 0,4
***18:2 n-6***	37,1 ± 5,3^a^	40,0 ± 7,8^ab^	49,7 ± 12,9^b^	16,8 ± 2,1	16,3 ± 2,1	15,9 ± 1,7
***20:0***	0,4 ± 0,0	0,4 ± 0,0	0,5 ± 0,1	0,2 ± 0,0	0,2 ± 0,0	0,1 ± 0,0
***18:3 n-6***	0,4 ± 0,1^a^	0,5 ± 0,1^a^	0,7 ± 0,2^b^	0,2 ± 0,0	0,2 ± 0,0	0,2 ± 0,1
***18:3 n-3***	1,2 ± 0,3	1,5 ± 0,5	2,1 ± 2,1	0,5 ± 0,1	0,6 ± 0,1	0,6 ± 0,4
***20:1***	1,7 ± 0,3^ab^	1,7 ± 0,4^a^	2,3 ± 0,7^b^	0,8 ± 0,1	0,7 ± 0,1	0,7 ± 0,1
***20:2***	1,5 ± 0,4^a^	1,6 ± 0,5^ab^	2,1 ± 0,7^b^	0,7 ± 0,2	0,6 ± 0,1	0,7 ± 0,2
***20:3 n-6***	0,5 ± 0,1^a^	0,6 ± 0,1^ab^	0,9 ± 0,3^b^	0,2 ± 0,0^a^	0,3 ± 0,0^ab^	0,3 ± 0,1^b^
***20:4 n-6***	1,2 ± 0,3^a^	1,8 ± 0,4^a^	2,3 ± 0,7^b^	0,6 ± 0,1^a^	0,7 ± 0,1^b^	0,7 ± 0,1^b^
***20:5 n-3***	0,2 ± 0,1^a^	0,3 ± 0,1^ab^	0,3 ± 0,1^b^	0,1 ± 0,0	0,1 ± 0,0	0,1 ± 0,0
***22:4 n-6***	2,1 ± 0,6^a^	2,0 ± 0,6^a^	3,5 ± 1,2^b^	0,9 ± 0,2	0,8 ± 0,2	1,1 ± 0,4
***22:5 n-6***	0,5 ± 0,1^a^	0,5 ± 0,1^ab^	0,7 ± 0,3^b^	0,2 ± 0,1	0,2 ± 0,0	0,2 ± 0,1
***22:5 n-3***	0,6 ± 0,2^a^	0,8 ± 0,3^ab^	1,2 ± 0,4^b^	0,3 ± 0,1	0,3 ± 0,1	0,4 ± 0,1
***22:6 n-3***	1,4 ± 0,5^a^	1,9 ± 0,8^ab^	2,7 ± 1,1^b^	0,6 ± 0,2	0,8 ± 0,3	0,9 ± 0,2
***unknown***	13,1 ± 3,3	14,9 ± 3,8	17,8 ± 6,0	-	-	-
***total***	221,7 ± 23,0^a^	245,6 ± 36,0^a^	315,5 ± 82,7^b^	-	-	-

Data are presented as means ± standard deviation. ^ab^Data with a different superscript within absolute or relative concentrations, differ significantly (P < 0.05). BMI: Body Mass Index, NEFA: Non-esterified fatty acid.

Absolute (μM) and relative (mol%) fatty acid concentrations in the phospholipid, cholesteryl ester, triglyceride and non-esterified fat fraction in the follicular fluid of normal weight (18.5 ≤ BMI < 25.0), overweight (25.0 ≤ BMI < 30.0) and obese (BMI ≥ 30.0) women undergoing assisted reproductive services.

#### Phospholipid fraction

The 4 most abundant FAs in the PL fraction of FF were 16:0, 18:0, 18:2 n-6 and 20:4 n-6 (Table 3), none of which were affected by BMI. On the contrary, the relative percentage of 18:1 n-9 was higher in normal weight women compared to overweight and obese women (*P* < 0.01) and the percentage of 20:3 n-6 was higher in overweight and obese women, compared to normal weight women (*P* = 0.04). There were no other BMI-related differences in FF FAs.

#### Cholesteryl-ester fraction

The most abundant FAs in this fraction were 16:0, 18:1 n-9, 18:2 n-6 and 20:4 n-6 (Table 3). Both the absolute and the relative concentration of 20:3 n-6 were significantly different between obese women and normal weight women (*P* = 0.04 and *P* = 0.02, respectively). There were no other BMI-related differences in the remaining FF FAs.

#### Triglyceride fraction

16:0, 18:0, 18:1 n-9 and 18:2 n-6 were major FAs in the TG fraction (Table 3). A significant elevation in the absolute concentration of 18:2 n-6 (*P* = 0.04) and 22:6 n-3 (*P* = 0.03) was found for overweight women, compared to normal weight women. There were no significant differences in the relative concentrations of the FAs.

#### Non-esterified fatty acid fraction

The most abundant FA is het NEFA fraction were 16:0, 18:0, 18:1 n-9 and 18:2 n-6 (Table 3). Absolute concentrations of 14:0, 15:0, 16:1 n-7, 16:1 n-9, 17:0, 18:0, 18:1 n-9, 18:1 n-11, 18:2 n-6, 20:2, 20:3 n-6, 20:5 n-3, 22:5 n-6, 22:5 n-3 and 22:6 n-3 were higher in obese compared to normal weight women (*P* < 0.05). Additionally, absolute concentrations of 16:0, 18:3 n-6, 20:4 n-6 and 22:4 n-6 were higher in obese compared to normal weight and overweight women (*P* < 0.05). Furthermore, obese women had higher concentrations of 20:1 compared to overweight women (*P* < 0.05). Relative concentrations only showed a significant elevation in 20:4 n-6 for overweight and obese women, compared to normal weight women (*P* < 0.05) and an elevation in 20:3 n-6 for obese women, compared to normal weight women (*P* < 0.01).

## Discussion

The aim of this study was to provide insight in the FA profile, specified per lipid fraction, in the FF of the pre-ovulatory follicle from normal weight, overweight and obese women, undergoing ART. To our knowledge, this is the first time that FAs in human FF lipid fractions are described in such detail. Our results showed that approximately 42% of the total FA concentration was esterified in the PL fraction and 34% in the CHE fraction, whereas only 10% were present in both the TG and NEFA fraction. Interestingly, only TG (trend) and NEFA concentrations were affected by BMI. Even though FA concentrations in the NEFA fraction were well below those of the other fractions, they showed the most BMI-related variability.

Total NEFA concentrations in the FF were elevated in obese women, which is confirmed by earlier work [18]. However, it is in contrast with our previous findings [17] and with the data of Robker *et al.*[35], who could not show a BMI-related difference in FF total NEFA concentrations. The difference between these data and those of our previous study, could partially be explained by a large variation in NEFA concentrations, the use of a different (colorimetric versus gas chromatographic) analysis methods and the inequality of the number of patients within each BMI group in our previous study [17]. Remarkably, our data also showed that even though absolute NEFA concentrations presented with a great BMI-related variability, relative concentrations only showed minor differences, suggesting that increasing BMI did not cause a shift in the relative abundance of the different FAs. Interestingly, Jungheim *et al.*[19] found that women with elevated levels of FF NEFAs displayed poorer cumulus-oocyte complex (COC) morphology. They differentiated between FF palmitic, stearic, oleic and linoleic acid in the NEFA fraction, but no correlations with BMI were found. Our data showed that 87% (20/23) of all FAs in the NEFA fraction, including palmitic, stearic, oleic and linoleic acid, correlated with BMI, which suggests that BMI causes changes in the FF NEFA concentrations that could directly affect COC function and quality. This is substantiated by the fact that we previously showed that bovine oocyte exposure, during the final stage of maturation, to elevated NEFA concentrations (oleic, palmitic and/or stearic acid) was detrimental for the oocyte’s developmental capacity [15]. It also altered gene expression patterns and energy/amino acid metabolism in blastocysts from oocytes exposed during the last 24 h of maturation [20]. Aardema *et al.*[36] showed that oocytes actively take up and metabolize NEFAs out of their environment (mitochondrial β-oxidation for the purpose of ATP production) and that this may influence lipid storage in lipid droplets within the oocyte, depending on the type and amount of FA offered. More specifically, oocytes exposed to palmitic and stearic acid presented with less intracellular fat storage and a hampered oocyte developmental competence. This effect could be counteracted by the addition of oleic acid to the treatment [36]. Such a protective effect of the mono-unsaturated FA oleic acid has also been proposed by Cnop et al. [37] in pancreatic islet cells, where oleic acid was described to reduce palmitate induced lipotoxicity, possibly by promoting triglyceride formation (cytoprotective mechanism). Our recent research investigated the mechanisms behind this lipotoxicity and showed that the degree of mitochondrial FA beta-oxidation has a strong impact on the development of NEFA exposed bovine oocytes and on the quality of the resulting embryos [21]. Besides the oocyte, the somatic cells of the follicle might also be influenced by the composition of FAs in the FF. For example, palmitic acid has been shown to inhibit *in vitro* bovine granulosa and theca cell proliferation and to alter steroid production by inducing apoptosis [38,39]. Arachidonic acid, on the other hand, protects human granulosa cells from palmitic and stearic acid-induced apoptosis [40]. The effects of poly-unsaturated FAs are less uniform as for example linoleic acid (18:2 n-6) hampers and linolenic acid (18:3 n-3) stimulates bovine nuclear oocyte maturation [6,8,41,42]. Interestingly, linoleic acid was present in the FF as one of the most predominant FAs. Marei et al. [41] showed that exposure of bovine cumulus-oocyte complexes to elevated linoleic acid concentrations during final maturation impaired oocyte maturation and decreased oocyte developmental competence. This may imply that elevated levels of linoleic acid could have harmful effects on fertility outcome in women. Under normal circumstances, linoleic acid concentrations are decreased in the FF of large follicles, compared to smaller follicles [43]. This suggest that linoleic acid can play an important role in the regulation of oocyte maturation, with decreasing levels allowing progression of oocyte maturation.

In all the previously discussed *in vitro* studies, FAs are added to the medium as non-esterified FAs. It remains, however, unclear if the same FAs, but esterified to a different fat fraction might elicit a differential effect. For example, a FA derived from the hydrolysis of TGs, contained in for example very low-density lipoproteins, by lipoprotein lipase [44], could potentially exert a different effect at the level of the cell, compared to the same FA in the NEFA fraction.

Besides the total NEFA concentrations, also total TG concentrations tended to be increased in overweight women. Interestingly, when mouse oocytes were matured in medium supplemented with NEFA/TG rich human FF, a dramatic decrease in oocyte maturation to metaphase 2, an increased oocyte lipid content and an upregulation of genes related to endoplasmic reticulum stress could be observed [18]. This suggests a detrimental effect of elevated TG and/or NEFA concentrations on oocyte quality. It is however not clear whether this is caused by elevated TG or NEFA concentrations or both or to what particular compound in the FF this effect can be attributed. Remarkably, 18:2 n-6 and 22:6 n-3 in the TG fraction were elevated in the FF of overweight, but not obese women, compared to normal weight women. A potential reason for this could be that in a first coping mechanism excess FAs, present in overweight individuals, are stored in lipid droplets [45]. However, in obese women, much of the circulating FAs originate from abdominal fat adipocytes [45], which are rich in saturated and mono-unsaturated FAs [46,47]. High levels of the saturated palmitic and stearic acid have been shown to reduce lipid storage in maturing bovine oocytes [36]. This is in agreement with our data, showing that obese women presented with elevated levels of many FA in the NEFA fraction, rather than in the TG fraction for potential storage in lipid droplets.

We have previously shown that a strong correlation exists between serum and FF metabolites, indicating that the serum composition influences the FF composition [17]. However, these changes were not BMI-related, indicating that factors, other than serum composition have a large impact on FF composition. Furthermore, the potential impact of differential FAs in the FF on oocyte developmental competence depends on the actual presence and the ratios of FAs within the FF. Therefore, the primary focus of our study was to describe FF FAs, rather than to investigate their relation to the serum composition. We furthermore did not have any knowledge on diet or fasting before sampling, even though it has been well described that diet can change the FF FA composition and can thereby affect oocyte developmental competence and subsequent embryo quality [48].

Another remark is that obese women required a higher dosage of gonadotropins to reach the same stimulatory effect on follicular development, compared to normal weight women. However, it has also been described that gonadotropin releasing hormone analogues can alter serum lipoprotein levels and can increase insulin resistance [49], potentially deteriorating the insulin resistant state in many obese women. Because of ethical restraints in proposing suboptimal gonadotropin concentration administration, we were unable to account for this potential confounding factor in our study.

## Conclusions

This descriptive study reports on the FA concentrations in the PL, CHE, TG and NEFA fraction in the FF of normal weight, overweight and obese women, undergoing ART. Our study highlights that most FAs in the FF belong to the PL and CHE fat fraction, but that NEFAs presented with the greatest BMI-related variability, with most individual FA concentrations increased in obese women. These differences may affect oocyte quality and subsequent embryo development, possibly by acting directly on oocyte metabolism.

## Abbreviations

2PN: Zygote presenting with 2 pronuclei; ART: Artificial reproductive techniques; BMI: Body mass index; CHE: Cholesteryl-ester; FA: Fatty acid; FF: Follicular fluid; NEFA: Non-esterified fatty acid; PL: Phospholipid; TG: Triglyceride.

## Competing interests

Valckx SDM declares that the study was funded by the special research fund, university of Antwerp (BOF UA). Fievez V and Vlaeminck B declare that the study was funded by the Fund for Scientific Research – Flanders (Belgium). The other authors have nothing to disclose.

## Authors’ contributions

SDMV, MAA and JLMRL contributed to the conception and design of the study, data analysis, interpretation of the data and drafting of the manuscript. IDP was responsible for patient recruitment, the collection of follicular fluid samples and aided to draft the manuscript. VF and BV carried out the gas chromatographic analyses and PEJB helped to draft the manuscript. All authors read and approved the final manuscript.
